# Downregulation of hepatic CYP3A isoforms by bardoxolone methyl in rats and its impact on *in vivo* metabolic capacity reflecting pharmacokinetics

**DOI:** 10.1038/s41598-026-50118-9

**Published:** 2026-04-28

**Authors:** Yuichiro Hori, Shuhei Fukuno, Hiroki Shirane, Rei Watanabe, Kyotaro Murata, Mei Takenaka, Hiroki Konishi, Katsuhito Nagai

**Affiliations:** Laboratory of Clinical Pharmacy and Therapeutics, Faculty of Pharmacy, Osaka Ohtani University, 3-11-1 Nishikiori-kita, Tondabayashi, 584-0066 Japan

**Keywords:** CYP3A isoform, Downregulation, Bardoxolone methyl, Nrf2 activator, Midazolam, Drug-drug interaction, Biochemistry, Diseases, Drug discovery, Gastroenterology, Medical research

## Abstract

Cytochrome P450 3A (CYP3A) enzymes metabolize many clinically important drugs. Previously, we clarified that bardoxolone methyl (BX), an Nrf2 activator, decreased CYP3A mRNA expression level in HepG2 cells. However, its effects on hepatic CYP3A protein and *in vivo* drug disposition remained unclear. This study investigated whether BX downregulates hepatic CYP3A expression and activity, and determine its impact on the pharmacokinetics of midazolam (MDZ), involving atypical substrate probing for CYP3A, in rats.　Male Sprague-Dawley rats received a single intraperitoneal dose of BX (10 mg/kg). BX decreased hepatic CYP3A mRNA and protein levels, and V_max_ value for MDZ hydroxylation without altering the K_m_ value. Intravenous MDZ pharmacokinetics (5 mg/kg) were unaffected following BX treatment; however, oral administration (15 mg/kg) showed reduced apparent clearance (CL_tot_/F), a measure of inherent *in vivo* metabolic capacity, leading to increases in AUC and bioavailability. No significant alterations in liver weight or plasma hepatic markers were observed. In conclusion, we suggest that hepatic CYP3A expression and intrinsic metabolic capacity are suppressed by BX, thereby markedly increasing the oral bioavailability of CYP3A substrates. These findings may help in the early detection of clinically significant drug interactions between Nrf2 activators and CYP3A substrate drugs.

**Supplementary Information:**

The online version contains supplementary material available at 10.1038/s41598-026-50118-9.

## Introduction

Hepatic cytochrome P450 3A (CYP3A) enzymes play central roles in not only biosynthesis and breakdown of endogenous substances required for the maintenance of physiological homeostasis but also oxidative metabolism of a wide array of xenobiotics including clinically important drugs, owing to its low substrate specificity and high expression level^1,2^. The metabolic capacity of hepatic CYP3A is susceptible to change by various factors, with drug interaction being recognized as a major cause. Considering that approximately 60% of drugs used in clinical settings undergo metabolic removal by CYP3A isoforms, evaluation of any change in hepatic CYP3A activity is of practical significance for predicting possible drug interactions. Midazolam (MDZ), a short-acting benzodiazepine, is widely used as a typical substrate probe for CYP3A activity in *in vitro* and *in vivo* studies^3,4^ because this agent is subject to metabolic conversion to 4-hydroxymidazolam (4-OH-MDZ) and α-hydroxymidazolam (α-OH-MDZ) by both CYP3A4 and CYP3A5 in humans^5^ and by both CYP3A1 and CYP3A2 in rats^6,7^, and it is almost completely eliminated from the body as polar metabolites^8^. Therefore, alterations in expressions or activities of CYP3A isoforms can influence the pharmacokinetics of MDZ and serve as an indicator of potential CYP3A-mediated drug interactions^9^. Drug interactions causing functional or quantitative reduction in CYP3A isoforms can lead to increased systemic exposure of co-administered drugs, resulting in developments of toxicity and therapeutic failure^10^. While several mechanisms have been proposed to explain these interactions, direct inhibition has been extensively studied for many drugs, including azole antifungals, macrolides, and synthetic steroids. However, transcriptional downregulation of CYP3A isoforms by low molecular weight compounds remains less well understood^11,12^.

Bardoxolone methyl (BX) is a semi-synthetic triterpenoid with the ability to improve the glomerular filtration rate under chronic renal impairment^13^. The pharmacological action of BX is considered to be based on activation of the nuclear factor erythroid 2-related factor 2 (Nrf2) pathway^14^. We previously demonstrated that expression of mRNA for CYP3A was significantly decreased in human hepatic carcinoma HepG2 cells exposed to BX^15^. This finding suggests that BX downregulates the CYP3A gene at the transcription level; however, it remains unknown whether expression of hepatic CYP3A protein and its enzyme activity are correspondingly decreased, and whether these changes reflect a decrease in the *in vivo* metabolic capacity that determines pharmacokinetic behavior of CYP3A substrate drugs.

The aim of the present study was to investigate the impact of BX on hepatic CYP3A expression and activity in rats, and assess its influence on the pharmacokinetics of MDZ following intravenous and oral administrations.

## Materials and methods

### Chemicals

Hydrochloride salt of MDZ and diazepam were purchased from Wako Pure Chemical Industries (Tokyo, Japan). BX, 4-OH-MDZ, and α-OH-MDZ were obtained from Sigma-Aldrich (St. Louis, MO, USA). Phenacetin was purchased from Cosmo Bio Co., Ltd. (Tokyo, Japan). All other reagents were of analytical or commercial grade and used without further purification.

### Animals

Male Sprague-Dawley rats (7 weeks old) were obtained from Japan SLC, Inc. (Hamamatsu, Japan). Rats were acclimatized for at least 2 days before assignment to experimental groups and housed in a clean room maintained at 23 ± 2 °C with 55 ± 10% relative humidity and a 12-h light/dark cycle. Animals had free access to standard chow and tap water. Rats were divided into two groups: BX-treated and control groups. In the BX group, rats received intraperitoneal BX (10 mg/kg, i.p.), and experiments were performed 48–96 h later. BX was dissolved in dimethyl sulfoxide. Control rats received vehicle alone with the same dosing volume. The vehicle volume was fixed at 0.4 µL per gram of body weight. The dose of BX was decided based on a previous report^16^.

The study protocol was approved by the Animal Experiment Committee of Osaka Ohtani University (ID number: 2404). All experimental procedures were performed in accordance with the relevant institutional guidelines and regulations. Rats were anesthetized with inhalational isoflurane (induction at 3–4%, maintenance at 1–2%) delivered *via* a nose cone. At the end of the study, euthanasia was performed by isoflurane overdose in accordance with institutional and international guidelines.

### Preparation of rat hepatic microsomes

Forty-eight hours after BX administration, rat livers were homogenized in three volumes of ice-cold 1.15% (w/v) potassium chloride solution using a Teflon homogenizer and centrifuged at 9,000 × *g* for 10 min. The supernatant was further centrifuged at 105,000 × *g* for 60 min to obtain microsomal pellets. Microsomal protein concentrations were determined by the Bradford method^17^ using bovine serum albumin (Wako Pure Chemical Industries) as the standard.

### Real-time quantitative PCR of CYP3A gene expression

Total RNA was extracted from the rat liver using ISOGEN (Wako Pure Chemical Industries) and purified with GenElute Mammalian Total RNA Miniprep Kit (Sigma-Aldrich). RNA was reverse-transcribed into cDNA using oligo (dT) primers and Moloney murine leukemia virus reverse transcriptase (GE Healthcare, Seattle, WA, USA). Gene expression levels were analyzed by real-time PCR using the CFX96™ system (Bio-Rad Laboratories, Berkeley, CA, USA) with SYBR Green dye (Toyobo Co., Ltd., Osaka, Japan). PCR amplification was performed at 95 °C for 10 s, 57 °C for 15 s, and 72 °C for 30 s. Melting curve analysis was conducted to confirm product specificity. Relative mRNA expression levels were quantified using the comparative cycle threshold method and normalized to GAPDH as the endogenous control. Primers were designed using Beacon Designer 8 (Bio-Rad). CYP3A1 was amplified with the 5’ primer 5’-TGCCATCACGGACACAGA-3’ and 3’ primer 5’-ATCTCTTCCACTCCTCATCCTTAG-3’. CYP3A2 was amplified with the 5’ primer 5’-GGACTTAATTGACTGCTCTTGATG-3’ and 3’ primer 5’-GGACGAGGACATGGTTAC-TATC-3’. GAPDH was amplified with the 5’ primer 5’-TTCAACGGCACAGTCAAG-3’ and 3’ primer 5’-TACTCAGCACCAGCATCA-3’. Results are expressed relative to control values (set as 1).

### Immunoblotting of CYP3A isoforms

Microsomal pellets were suspended in lysis buffer (50 mM Tris-HCl, pH 7.5, 150 mM NaCl, 0.5% Nonidet P-40, and protease inhibitor cocktail (Nacalai Tesque Inc., Kyoto, Japan)). Protein samples (10 µg) were separated by SDS-PAGE and transferred to PVDF membranes (Bio-Rad). Membranes were washed with PBS containing 0.1% Tween-20 (PBS-T) and blocked with Blocking One (Nacalai Tesque Inc.) for 1 h at room temperature. After washing, membranes were incubated with rabbit anti-CYP3A1 (GeneTex Inc., Irvine, CA, USA; 1:1000) or rabbit anti-CYP3A2 antibodies (Santa Cruz Biotechnology, Dallas, TX, USA; 1:1000) for 1 h at room temperature, followed by HRP-conjugated anti-rabbit IgG (GE Healthcare; 1:5000) for 1 h. Immunoreactive bands were visualized using ECL reagent (Wako Pure Chemical Industries). PVDF membranes were subsequently stained with Coomassie Brilliant Blue (CBB). Band intensities were quantified using CSAnalyzer software (Atto Corp., Tokyo, Japan). Data are expressed relative to control values (set as 1).

### Measurement of MDZ hydroxylation activity in rat liver microsomes

Microsomal pellets were resuspended in 80 mM sodium/potassium phosphate buffer (pH 7.4). A mixture for enzyme reactions (500 µL) contained an NADPH-generating system (0.5 mM NADP^+^, 5 mM glucose-6-phosphate, 5 mM MgCl₂, 2 units glucose-6-phosphate dehydrogenase, 1 mM EDTA), 80 mM phosphate buffer, and MDZ (2.5–100 µM) with liver microsomes (1 mg/mL). The enzyme reactions were initiated by adding MDZ and incubating at 37 °C for 5 min with constant shaking. For inhibition studies, untreated microsomes were used. BX (0–50 µM) was added to a reaction mixture to evaluate its inhibitory effects on MDZ 4-hydroxylation and α-hydroxylation in the presence of MDZ (25 µM). Reactions were terminated by adding 200 µL of 0.2 M sodium hydroxide.

### MDZ administration and blood sampling

MDZ administration and blood sampling were performed 48 h and 96 h after BX administration. The right and left jugular veins were cannulated with polyethylene tubing (Natsume Seisakusyo Co., Ltd., Tokyo, Japan) under anesthesia 1 day prior to pharmacokinetic studies. Tubes were exteriorized to the interscapular area. Rats were allowed to recover overnight. MDZ was dissolved in 0.9% sodium chloride solution (pH adjusted to 3.5) and administered intragastrically (15 mg/kg) or intravenously *via* the right jugular vein (5 mg/kg), according to previous studies^18,19^. Serial blood samples (250 µL) were collected from the left jugular vein for up to 180 (i.v.) or 240 (p.o.) min. Heparinized saline was used to maintain catheter patency between samplings. Plasma was obtained by centrifugation of blood (10 min).

### Measurement of metabolites and plasma MDZ concentrations

4-OH-MDZ and α-OH-MDZ, CYP3A-dependent metabolites of MDZ, were measured according to the report of Elkiran et al.^20^. After termination of reactions, 50 µL of 2 µM phenacetin (internal standard: IS) and 4 mL diethyl ether were added. Samples were shaken for 10 min and centrifuged at 3,000 rpm for 10 min at 4 °C. The organic layer was evaporated at 50 °C, and the residue was reconstituted in a 200-µL mobile phase. A 100-µL aliquot was injected into an HPLC system (LC-2050C 3D Plus, Shimadzu, Kyoto, Japan) using an RP-18 GP II column (5 μm, 150 × 4.6 mm). The mobile phase consisted of acetonitrile/10 mM phosphate buffer (pH 7.4)/methanol (35:60:5, v/v/v) at 1.0 mL/min, with detection at 214 nm. Plasma MDZ concentrations were determined as previously reported^21^. Plasma (100 µL) was mixed with IS (50 µL of 20 µg/mL phenacetin) and 200 µL of 0.1 M sodium hydroxide, and then extracted with 4 mL of diethyl ether. After centrifugation (3,000 × *g*, 10 min, 4 °C), the organic layer was evaporated at 50 °C. The residue was dissolved in a 200-µL mobile phase, and 100 µL was injected into the HPLC system. Separation was achieved with an RP-18 GP II column (5 μm, 150 × 4.6 mm) using a mobile phase of 10 mM phosphate buffer (pH 4.4)/acetonitrile (62:38, v/v) at 1.0 mL/min, 40 °C, with detection at 220 nm.

### Kinetic analysis of MDZ hydroxylation

According to previous studies^18,19^, kinetic analyses of MDZ 4-hydroxylation and α-hydroxylation were performed with the consideration that enzyme reactions obeyed the standard Michaelis-Menten model. Sets of data points were subjected to the following equation to estimate apparent Michaelis-Menten kinetic parameters: V = V_max_ × S/(K_m_ + S), where V, V_max_, S, and K_m_ are the velocity of the reaction, maximum reaction velocity, MDZ concentration, and Michaelis constant, respectively. Individual parameters were calculated by the non-linear least-squares curve-fitting method using GraphPad Prism 10 (GraphPad Software, Inc., San Diego, CA, USA).

### Pharmacokinetic analysis of MDZ

Pharmacokinetic parameters were calculated using non-compartmental analysis. The terminal elimination rate constant (λ_z_) was obtained by linear regression of the terminal log-linear concentration–time profile. The area under the curve (AUC_0→∞_) was calculated using the linear trapezoidal rule with extrapolation to infinity (Cp_(last)_/λ_z_). Total clearance (CL_tot_) and apparent clearance (CL_tot_/F) were obtained by dividing the dose by AUC_0→∞_. The area under the first-moment curve (AUMC) was calculated with a similar approach, with extrapolation defined as t_(last)_ × Cp_(last)_/λ_z_ + Cp_(last)_/(λ_z_)². Mean residence time (MRT) was calculated as AUMC/AUC_0→∞_. The apparent steady-state volume of distribution (Vd_ss_) was determined as CL_tot_ × MRT.

### BX administration and measurement of its plasma concentration

Plasma samples were collected 48 h after intraperitoneal administration of BX to rats at a dose of 10 mg/kg. Plasma BX levels were quantified using an absolute calibration method based on a previously reported procedure with minor modifications^22^. Briefly, 7 mL of *tert*-butyl methyl ether was added to 200 µL of plasma, and the mixtures were shaken for 10 min followed by centrifugation at 3,000 rpm for 10 min at 4 °C. The organic layer was evaporated at 50 °C. The resulting residue was reconstituted in 200 µL of the mobile phase, and a 100-µL aliquot was injected into the HPLC system. The mobile phase consisted of a gradient elusion from acetonitrile/25 mM acetate buffer (pH 3.75) at a ratio of 80:20 (v/v) to 95:5 (v/v) over 8 min, with a flow rate of 1.0 mL/min and detection at 238 nm.

### Statistical analysis

Data are expressed as means ± SD. Differences between the means of two groups were evaluated using Student’s unpaired *t-*test (two-tailed). The significance of differences between control and test values was determined using Dunnett’s test (two-tailed). Differences with a *p*-value of 0.05 or less were considered significant.

## Results

### Effects of BX on expressions of CYP3A1 and CYP3A2

**Fig. 1:**
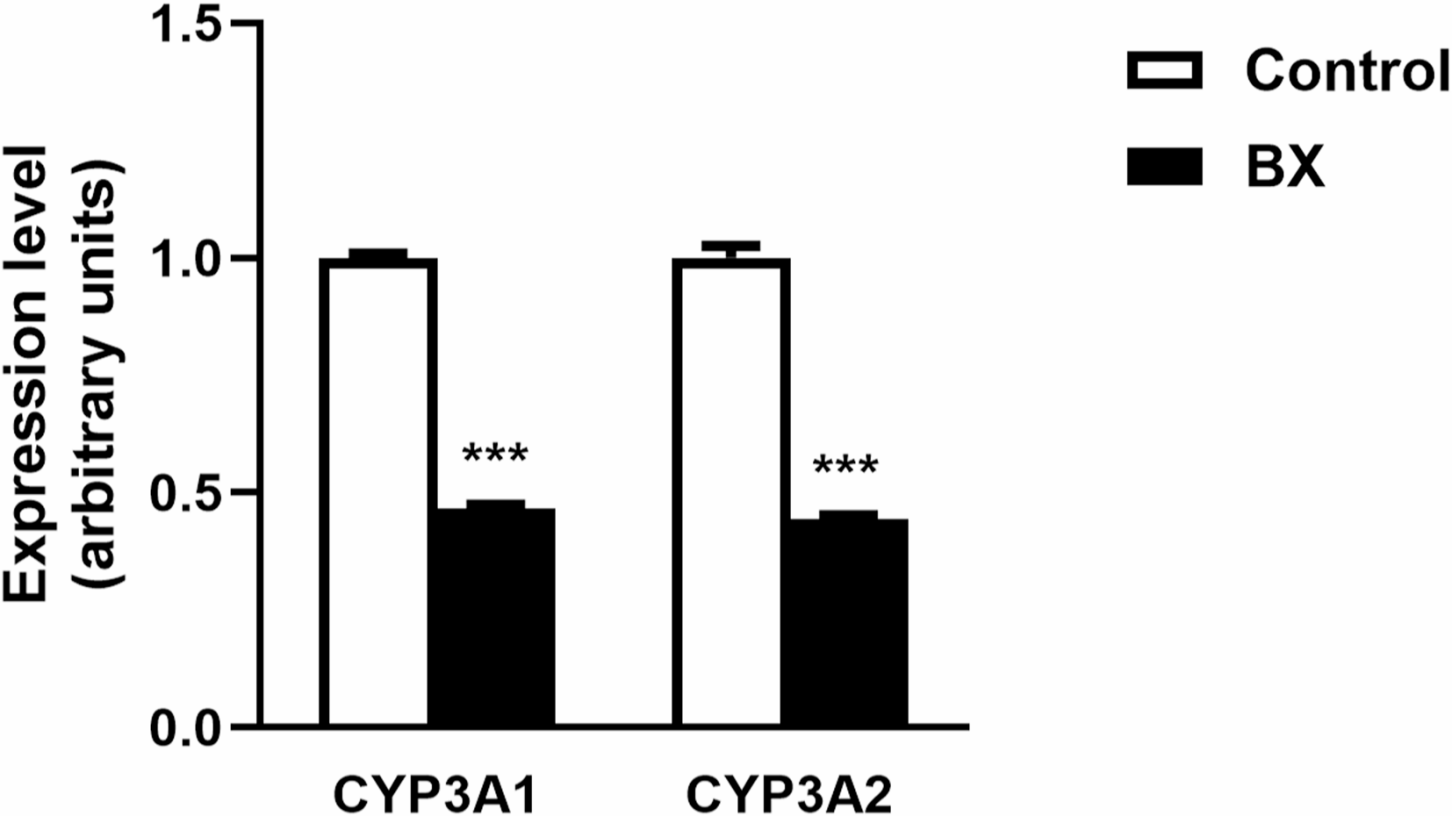
Hepatic mRNA expression levels of CYP3A1 and CYP3A2 in control and BX-treated rats. Results are expressed as the mean ± SD of three rats per group. ***Significantly different from the control group (*p* < 0.001, Student’s unpaired *t*-test).

**Fig. 2:**
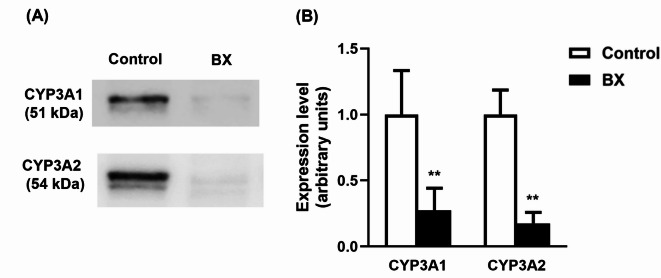
Hepatic protein expression levels of CYP3A1 and CYP3A2 in control and BX-treated rats. Calculated values for visualized immunoreactive bands (**A**) are shown as the mean ± SD of four rats per group (**B**). **Significantly different from the control group (*p* < 0.01, Student’s unpaired *t*-test).

We used real-time PCR and *Western* blot analysis to examine whether hepatic expressions of mRNAs and proteins for CYP3A isoforms were altered in the BX-treated rats (Figs. 1 and 2). Expression levels of mRNA and protein for both CYP3A isoforms decreased by more than 50% in rats treated with BX (CYP3A1 and CYP3A2 mRNA: *p* < 0.0001; CYP3A1 protein: *p* = 0.0080; CYP3A2 protein: *p* = 0.0002; two-tailed unpaired *t*-test). CBB-stained images of proteins transferred onto the PVDF membrane were similar in groups (data not shown).

### Enzyme kinetics of MDZ 4- and α-hydroxylation after BX exposure

**Fig. 3:**
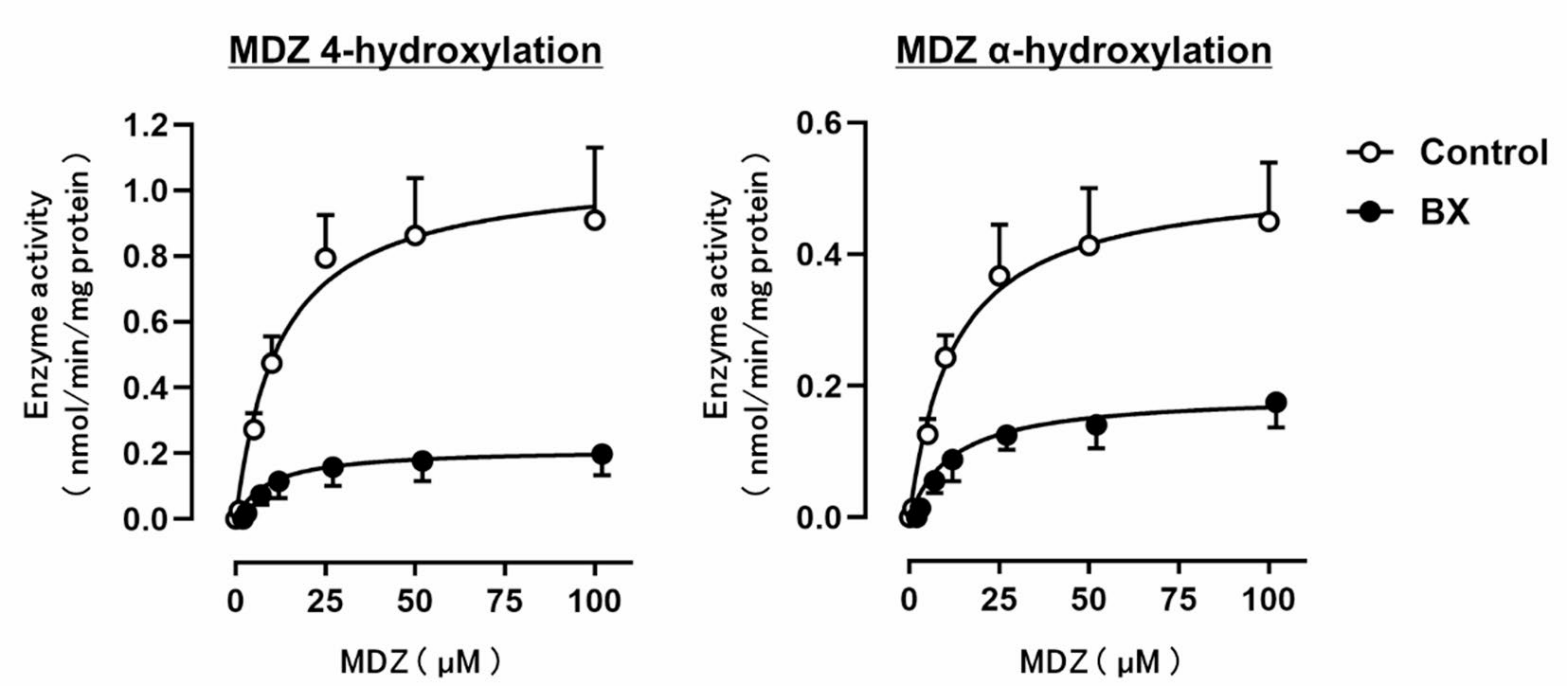
Enzyme kinetics of MDZ 4-hydroxylation and α-hydroxylation in rat liver microsomes of control and BX-treated rats. MDZ was reacted with 1 mg/mL of microsomes for 5 min at 37 °C. The results are shown as the mean ± SD of six rats per group.

Microsomal enzyme activities of the 4- and α-hydroxylation of MDZ were measured in BX-treated and control rats to assess the direct effects of BX on the hepatic CYP3A function. Michaelis-Menten plots for enzyme reactions by CYP3A isoforms showed that enzyme activity in the BX-treated group was lower than in the control group (Fig. 3). The V_max_ values in the BX-treated group estimated by these plots were significantly lower than in the control group (4-hydroxylation: *p* = 0.0496; α-hydroxylation: *p* = 0.0157; two-tailed unpaired *t*-test), while there was no significant difference in K_m_ values between the BX-treated and control groups (Table 1).

**Table 1:** Effects of BX treatment on MDZ 4-hydroxylation and α-hydroxylation in rat liver microsomes.

	MDZ 4-hydroxylation		MDZ α-hydroxylation	
	Control	BX	Control	BX
V_max_ (nmol/min/mg protein)	1.07 ± 0.24	0.22 ± 0.06 ^***^	0.52 ± 0.11	0.19 ± 0.03 ^***^
K_m_ (µM)	12.1 ± 2.6	9.7 ± 1.7	12.4 ± 2.2	13.0 ± 4.2
V_max_/K_m_ (µL/min/mg protein)	89.2 ± 11.8	23.8 ± 10.9 ^***^	42.3 ± 6.5	15.8 ± 5.0 ^***^
The results are shown as the mean ± SD of six per group. ***Significantly different from the control group (*p* < 0.001, Student’s unpaired *t*-test).				

### Direct inhibition of MDZ 4- and α-hydroxylation by BX

**Fig. 4:**
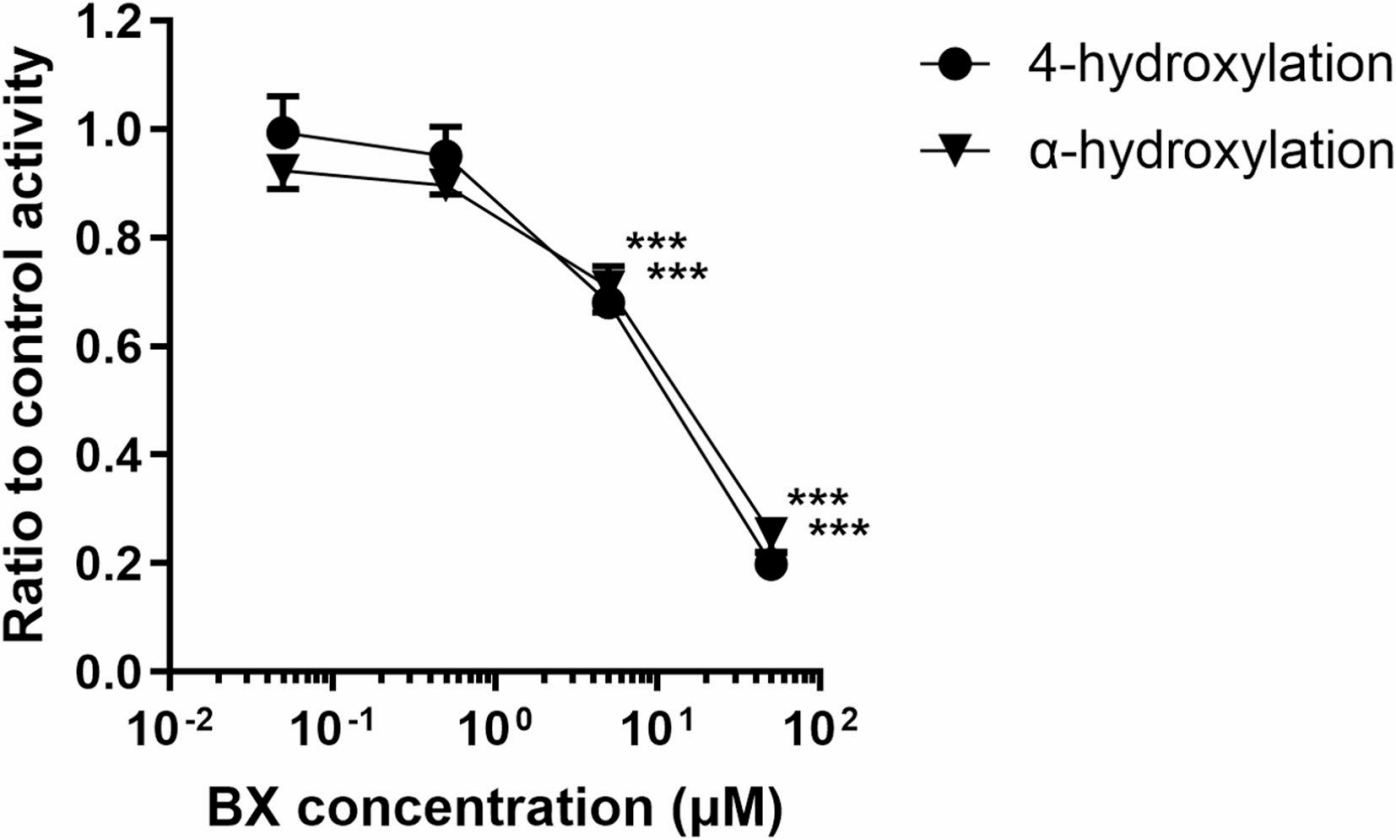
*In vitro* effects of BX on MDZ 4-hydroxylation and α-hydroxylation in untreated rat liver microsomes. MDZ was reacted with microsomes in the presence of BX (0–50 µM) at 37 °C using an NADPH-regenerating system. BX concentrations are presented in a logarithmic scale. Data are expressed as the ratio to activity measured without BX. The results are shown as the mean ± SD of four rats per group. ***Significantly different from the BX 0 µM group (*p* < 0.001, Dunnett’s test).

As shown in Fig. 4, the microsomal enzyme activities of MDZ 4- and α-hydroxylation were decreased BX concentration-dependently, but the degree of decrease was minor, and significant inhibition was observed only at BX concentrations exceeding 5 µM (4-hydroxylation, 0 µM vs. 5 µM: *p* = 0.0002; 4-hydroxylation, 0 µM vs. 50 µM, α-hydroxylation, 0 µM vs. 5 µM and 0 µM vs. 50 µM: *p* < 0.0001; Dunnett’s test).

### Effects of BX on body weight and hepatic function

We evaluated the effects of BX treatment on hepatic markers in plasma, and body and liver weights 48 and 96 h after administration (Table 2). BX had no significant effect on body or liver weights, and there were no significant differences in plasma activities of AST or ALT or the concentration of albumin between BX-treated and control groups.

**Table 2:** Body and liver weights and hepatic markers of control and BX-treated rats.

	48 h after administration		96 h after administration	
	Control	BX	Control	BX
Body weight (g)	245 ± 4	241 ± 5	245 ± 15	238 ± 12
Liver weight (g)	13.6 ± 1.4	13.6 ± 1.6	13.2 ± 1.6	13.9 ± 2.0
Liver weight ratio (%)	5.49 ± 0.61	5.62 ± 0.59	5.41 ± 0.58	5.80 ± 0.66
ALT (U/L)	34.0 ± 21.0	36.9 ± 16.6	28.6 ± 11.8	29.6 ± 11.7
Albumin (g/dL)	2.85 ± 0.24	2.87 ± 0.26	3.00 ± 0.29	3.11 ± 0.12
The results are shown as the mean ± SD of ten rats per group.				

### Plasma BX concentration

Plasma BX concentration 48 h after intraperitoneal administration was 0.025 µM.

### Pharmacokinetics of MDZ after intravenous and oral administrations

**Fig. 5:**
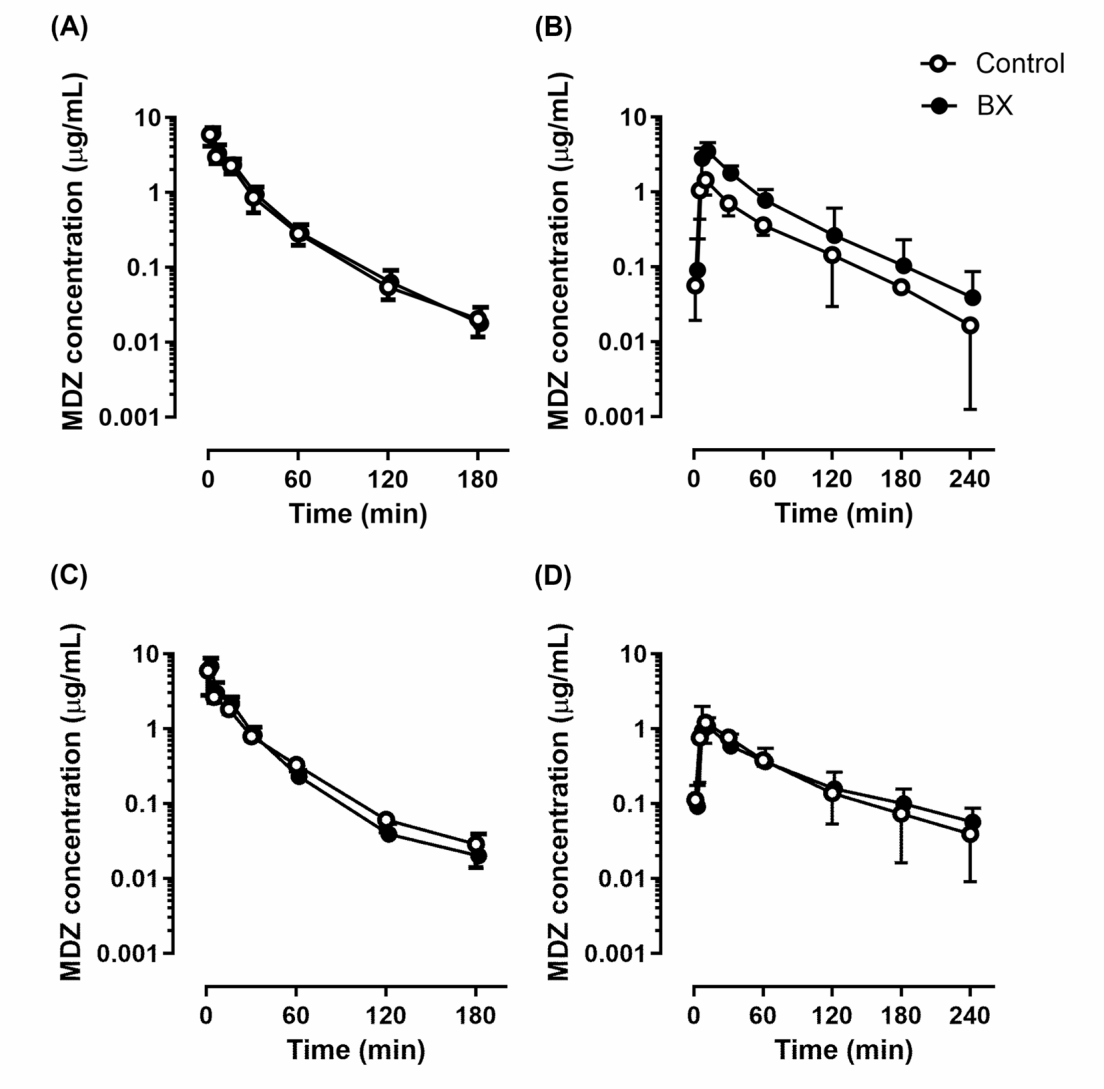
Plasma concentration-time course of MDZ after its intravenous (5 mg/kg, A and C) or oral (15 mg/kg, B and D) administration to control and BX-treated rats. The examinations were conducted 48 h (A and B) or 96 h (C and D) after the administration of BX or vehicle alone. Serum MDZ concentrations are presented in a logarithmic scale. The results are shown as the mean ± SD of five rats per group.

The plasma concentration-time profiles of MDZ following intravenous and oral administrations to BX-treated and control rats are shown in Fig. 5. There was no significant difference in plasma MDZ concentrations at each sampling point following intravenous administration between BX-treated and control groups (Fig. 5A). However, 48 h after BX administration, the plasma MDZ concentrations after oral administration were higher in BX-treated than control rats (Fig. 5B). The relevant pharmacokinetic parameters estimated using the non-compartment method are also listed in Table 3. In the case of intravenous administration, BX treatment had no effect on any of the pharmacokinetic parameters estimated by plasma concentration-time profiles of MDZ. The values of AUC (*p* = 0.0011; two-tailed unpaired *t*-test) and F (*p* = 0.0030; two-tailed umpaired *t*-test) in the BX-treated rats orally administered MDZ were significantly higher than in the control group, while CL_tot_/F was significantly lower in the BX-treated than control group (*p* = 0.0018; two-tailed umpaired *t*-test). In contrast, 96 h after BX administration, no significant differences in the pharmacokinetics of MDZ were observed between two groups, regardless of its administration route (Fig. 5C and 5D, and Table 3).

**Table 3:** Pharmacokinetic parameters of MDZ after intravenous or oral administration to control and BX-treated rats.

	48 h after administraion						96 h after administration						
	Control			BX			Control			BX			
Intravenous administration (*n* = 5)													
AUC_0→∞_ (mg·h/L)	1.67 ± 0.29			1.56 ± 0.41			1.48 ± 0.20			1.54 ± 0.30			
CL_tot_ (L/kg/h)	3.08 ± 0.46			3.45 ± 0.96			3.43 ± 0.46			3.35 ± 0.53			
λ_z_ (1/h)	1.42 ± 0.30			1.46 ± 0.30			1.09 ± 0.43			1.27 ± 0.35			
Vd_ss_ (L/kg)	1.52 ± 0.33			1.34 ± 0.35			1.86 ± 0.47			1.42 ± 0.52			
Oral administration (*n* = 5)													
AUC_0→∞_ (mg·h/L)	1.22 ± 0.27			2.68 ± 0.53**			1.20 ± 0.29			1.27 ± 0.21			
CL_tot_/F (L/kg/h)	12.9 ± 2.9			5.81 ± 1.09**			13.2 ± 3.1			12.2 ± 2.1			
λ_z_ (1/h)	1.23 ± 0.64			0.98 ± 0.35			0.72 ± 0.15			0.57 ± 0.31			
F (%)	25.1 ± 2.1			61.6 ± 6.7**			27.5 ± 2.4			28.3 ± 2.1			
The results are shown as the mean ± SD of five rats per group, except that the F value is shown as the mean ± SE. **Significantly different from the control group (*p* < 0.01).													

## Discussion

In current clinical practice, pharmacotherapy is conducted frequently by combining several types of medications, and it is necessary to pay attention to drug interactions responsible for unexpected changes in therapeutic efficacy or the occurrence of adverse events. In the present study, we examined the effects of BX on the hepatic expression and activity of CYP3A isoforms in rats, and on the pharmacokinetics of MDZ administered intravenously and orally.

In agreement with the previous findings obtained using a cultured cell line^15^, the decreased expressions of mRNA for CYP3A isoforms were also confirmed *in vivo* using rats treated with BX. Correspondingly, a significant decrease in hepatic expressions of proteins for CYP3A1 and CYP3A2 was also demonstrated in the translation stage. This finding was supported by the results of enzyme kinetic analysis showing significantly lower V_max_ values for both MDZ 4- and α-hydroxylation reactions, which are catalyzed primarily by CYP3A^7^, in the BX-treated group compared with those in the control group.

It has been reported that intraperitoneal administration of BX (2–10 mg) to rats activates the Nrf2 signaling pathway and elicits potent antioxidant and anti-apoptotic effects within 24 hours^15,23^. The Nrf2 pathway is a master regulator of cellular defense against oxidative stress and xenobiotic-induced damage, and controls the expression of numerous cytoprotective genes, including antioxidant enzymes (HO-1, NQO1) and phase II detoxification enzymes, by binding to antioxidant response elements in their promoters^24^. Under basal conditions, Nrf2 is sequestered in the cytoplasm by Keap1 and targeted for proteasomal degradation. Upon oxidative or electrophilic stress, Nrf2 dissociates from Keap1, translocates to the nucleus, and initiates the transcription of genes that restore redox homeostasis. Taking the diversity of these signaling networks into account, BX may enable modulation of various genes, including CYP3A isoforms in the liver, as shown in the present study. However, the detailed mechanisms underlying regulation of the gene expression of hepatic CYP3A isoforms following BX administration remain unclear, and further investigation will be required.

To investigate whether the decreased expression of hepatic CYP3A isoforms reflects the *in vivo* metabolic capacity, we evaluated the pharmacokinetic behavior of MDZ. Following intravenous administration of MDZ, the plasma concentration-time profile remained comparable with that of the control group even on BX exposure, and no significant changes were observed in any pharmacokinetic parameters. A likely explanation for these observations is that the hepatic clearance of MDZ after entering the systemic circulation is governed mainly by blood flow in the liver, a property common to intravenously administered drugs with a high hepatic extraction ratio. The absence of any significant change in CL_tot_ suggests that BX does not cause hepatotoxicity that impairs hepatic blood flow, being consistent with the lack of deterioration of hepatic markers and liver weight. When MDZ was administered orally, its plasma concentrations were markedly elevated on the 48 h after BX administration. However, no change was observed in the plasma concentration of MDZ on day 4, suggesting that the effects of BX causing alteration in the MDZ disposition had disappeared at this point. Pharmacokinetic analysis revealed that AUC in the BX-treated group on day 2 was significantly higher than in the control group, whereas the apparent total clearance of CL_tot_/F was significantly reduced. In contrast to the CL_tot_ value, the CL_tot_/F value of drugs undergoing high hepatic extraction is dependent upon their hepatic intrinsic clearance (CL_int_) and protein-binding rate in plasma rather than hepatic blood flow. Regarding these two parameters, the possibility of changes in the protein-binding rate can be ruled out since there were no significant differences in plasma albumin levels or Vd_ss_ between the control and BX-treated groups. The metabolic clearance of MDZ accounts for most of its elimination capacity and, thereby, can be approximated to total clearance. In addition, hepatic metabolism contributes approximately 85% to the first-pass removal of MDZ despite its high intestinal absorbability, with rapid and marked permeation across gastrointestinal epithelial cells^25^. Moreover, although MDZ is regarded as a substrate of P-glycoprotein^26^, its overall contribution appears to be negligible as apical uptake of MDZ to enterocytes is virtually unaffected by P-glycoprotein inhibitors in rats^27^. Therefore, the decreased CL_tot_/F value is considered attributable to a reduction in CL_int_, which explains the increased bioavailability. The bioavailability of drugs undergoing extensive first-pass metabolism in the liver is theoretically altered in response to the intrinsic metabolizing capacity of hepatic enzymes when administered orally, and these characteristics hold true for most CYP3A substrate drugs including MDZ. In this study, the intrinsic clearance represents inherent CYP3A activity to metabolically eliminate MDZ without being affected by other external factors. According to the well stirred model, which assumes that drugs entering the hepatic blood vessels are immediately mixed, resulting in a uniform concentration in the liver, the rate of metabolic removal of drugs corresponds to the product of the CL_int_ and plasma unbound concentration. Therefore, the V_max_/K_m_ values calculated from enzyme kinetic analysis provide a good assessment of CL_int_ as an unbound concentration of MDZ available for metabolism being lower than the K_m_ values, given the high serum protein binding rate of MDZ (> 95%)^28^. On the other hand, the pharmacokinetics of BX in rats have not been clarified; however, the plasma BX concentration 48 h after intraperitoneal administration (0.025 µM) was two orders of magnitude lower than the concentration required to significantly inhibit MDZ hydroxylation in hepatic microsomes, strongly suggesting that a decrease in CL_int_ by direct inhibition of CYP3A isoforms by BX is unlikely. These results demonstrate that BX potently downregulates hepatic CYP3A isoforms, and that decreased enzyme activities reflect decreases in the *in vivo* capacity to metabolize their substrates.

In clinical and experimental studies, many cases have been reported in which gene modulation for CYP3A isoforms induced pharmacokinetic interactions, but the mechanism of most of these observations was based on CYP3A upregulation^29^. Although several studies reported decreased expression of CYP3A under specific physiological conditions such as cancer^30^, chronic renal insufficiency^31^, systemic inflammatory disorders^32^, and severe infectious diseases^33^, these events do not describe interactions caused by concomitant medications. It has been reported that Nrf2 deficiency altered expression levels of several CYPs in mice^34^, which leads to the interpretation that activation of the Nrf2-mediated signaling pathway is likely to be the molecular mechanism causing altered expression levels of CYP3A isoforms by BX. In the present study, we demonstrated using rats that BX regulates gene expression for CYP3A, probably by activation of Nrf2, and consequently alters the pharmacokinetics of CYP3A substrate drugs due to the decreased intrinsic hepatic clearance. This is the first study to provide fundamental evidence for the downregulation of CYP3A isoforms leading to *in vivo* pharmacokinetic drug-drug interactions. Species-specificity is known to exist in the profile of CYP3A expression. While there is little sex difference in humans, CYP3A2 is specifically expressed only in male rats^35^. However, the expression of CYP3A enzymes is concurrently regulated through a common pathway involving the pregnane X receptor (PXR) and the constitutive androstane receptor across many species. We previously demonstrated decreased mRNA expression for CYP3A4 in human HepG2 cells^15^. This finding underscores the translational relevance of the present findings obtained using rats, and suggests that similar types of drug-interactions may occur in humans. Given the ongoing clinical development of BX for chronic kidney disease and related disorders, clinicians should anticipate potential drug-drug interactions with CYP3A substrates during early treatment phases, even if no interactions have been reported to date. Incorporating human *in vitro* data and clinical evidence will strengthen the rationale for proactive monitoring and individualized dosing strategies. The present study focused on CYP3A in the liver, therefore, changes in the morphology and metabolic function of the small intestine were not evaluated. Further research should include the molecular analyses of CYP3A expression post-BX exposure, evaluation of intestinal CYP3A-mediated metabolism, and clinical pharmacokinetic studies in patients receiving BX in combination with CYP3A substrates.

The present study provides preclinical evidence for the regulatory effect of BX on hepatic CYP3A expression. Sulforaphane, which activates Nrf2 through modification of Keap1 cysteine residues similarly to BX, has been reported to reduce the metabolic capacity of CYP3A in healthy adults, with this alteration being particularly pronounced in the subset with high CYP3A activity level^36^. Concordant results were obtained in humanized PXR mice[^36^]. In contrast, dimethyl fumarate, a clinically used Nrf2 activator, has been shown not to exert direct inhibitory or inductive effects on CYP3A activity[^37^], and to date, no clinically apparent drug-drug interactions have been reported. However, with respect to CYP3A downregulation in view of gene expression, available evidence remains insufficient to conclude that each individual compound has been adequately evaluated. Taken together, while differences in the regulatory effects on CYP3A among Nrf2 activators should be considered, our findings suggest that, when Nrf2 activators are used in clinical practice, it is prudent to avoid premature interpretation and to carefully assess the potential for interactions with CYP3A substrate drugs.

In conclusion, we demonstrated that BX increases MDZ oral bioavailability by reducing pre-systemic metabolism through CYP3A downregulation. The interaction arising from gene downregulation may require particular attention in terms of severity because irreversible depression of CYP3A activities will persist even after BX disappears from the body. These findings may help in the early detection of clinically significant drug interactions between Nrf2 activators and CYP3A substrate drugs.

## Supplementary Information

Below is the link to the electronic supplementary material.

Supplementary Material 1

## Data Availability

The data that support the findings of this research can be obtained from the corresponding author upon reasonable request.
