# Supplementary figures and images for: Downregulation of hepatic CYP3A isoforms by bardoxolone methyl in rats and its impact on in vivo metabolic capacity reflecting pharmacokinetics
Source: Sci Rep. 2026 Apr 28;16:20178. doi: 10.1038/s41598-026-50118-9 (PMC13324016; doi:10.1038/s41598-026-50118-9)

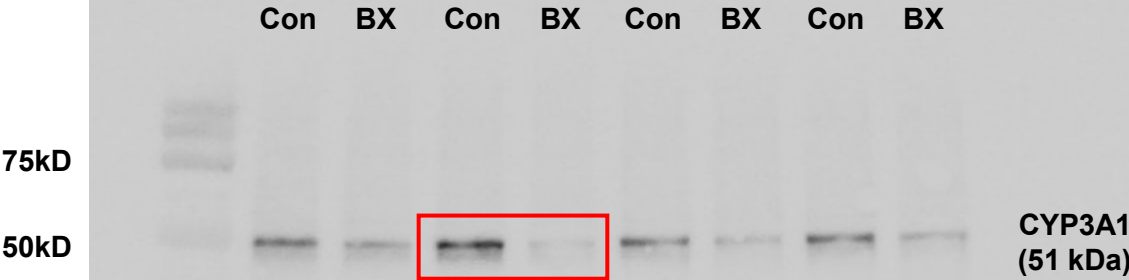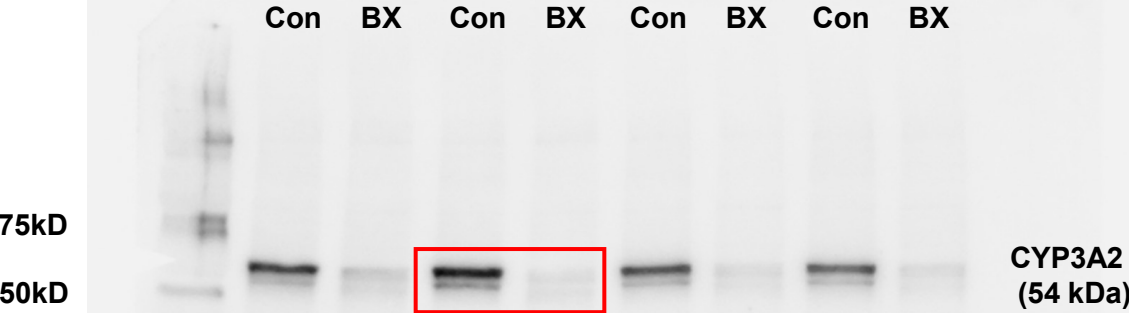

Supplement: Supplementary file 1 — Supplementary Material 1 [file 41598_2026_50118_MOESM1_ESM.pdf]
